# Proteomic Analysis of patients with Epileptic Seizure and Psychogenic Non-epileptic Seizure; a Cross-Sectional Study

**Published:** 2020-03-11

**Authors:** Mohsen Parvareshi Hamrah, Mostafa Rezaei Tavirani, Monireh Movahedi, Sanaz Ahmadi Karvigh

**Affiliations:** 1 Department of Biochemistry, Faculty of Biological Science, North Tehran Branch, Islamic Azad University, Tehran, Iran.; 2 Proteomics Research Center, School of Allied Medical Sciences, Shahid Beheshti University of Medical Sciences, Tehran, Iran (https://orcid.org/0000-0003-1767-7475).; 3 Department of Neurology, Sina Hospital, Tehran University of Medical Sciences, Tehran, Iran.

**Keywords:** Seizures, proteomics, biomarkers, diagnosis, differential

## Abstract

**Introduction:**

There is an increasing interest in the use of different biomarkers to help distinguish psychogenic non-epileptic seizure (PNES) from epileptic seizures (ES). This study aimed to evaluate the patterns of differentially expressed serum proteins in ES and PNES cases.

**Methods:**

In this cross-sectional study, 4 patients with mesial temporal lobe epilepsy and 4 patients with PNES were selected from patients with history of recurrent seizures. Venous blood samples were obtained within 1 hour after seizure and serum proteomes as well as the extent of protein expression were analyzed.

**Results:**

361 proteins were identified; of these, expression of 197 proteins had altered. 110 (55.9%) proteins were down-regulated and 87 (44.1%) were up-regulated in the PNES samples compared to ES samples. The mean pI for deregulated proteins with 1.5 to 3 fold changes were 6.69 ± 1.68 in proteins with increasing expression in ES group and 5.88 ± 1.39 in proteins with increasing expression in PNES group (p = 0.008). The median and interquartile range (IQR) of molecular weight changes in proteins with 1.5 to 3 fold changes were 64 (22.0-86.0) in proteins whose expression had increased in ES group and 39.5 (26.0-61.5) in proteins whose expression had increased in PNES cases (p = 0.05).

**Conclusion:**

Several spots with differential expression were observed by comparing patients with ES against the PNES groups, which could be potential biomarkers of the disease. Damage to the blood-brain barrier is the most important difference between the two groups, thus identifying total protein changes offers a key to the future of differentiating ES and PNES patients.

## Introduction

Psychogenic non-epileptic seizures (PNES) are neither paroxysmal behavioral changes resembling epileptic seizures (ES) without organic cause nor ictal, peri-ictal, and inter-ictal electroencephalography (EEG) changes (1). Approximately 80% of patients with video-EEG (VEEG) confirmed PNES were taking at least one antiepileptic drug (AED) at the time of diagnosis (2). About 5–20% of patients presenting to an epilepsy unit for VEEG monitoring are diagnosed with PNES, and 20–30% of intractable seizures are finally diagnosed as PNES (3). Misdiagnosis leads to many years of wrong treatment regimens, experiencing side effects of drugs, additional financial burdens, and adverse effects on social life (4-6). The gold standard for diagnosis is the recording of a typical event with VEEG to confirm the absence of electrographic changes on the ictal phase. Although VEEG monitoring is the gold standard for diagnosis of PNES, it has the limitations of high cost, low accessibility, and long hospitalization. Moreover, the habitual events sometimes may not be captured during monitoring. Considering the mentioned limitations, other diagnostic tests have been applied to help differentiate ES from PNES. 

Comparative proteomic analysis is a powerful diagnostic tool to determine the onset, progression, and prognosis of human diseases. It can identify a large number of proteins simultaneously, and protein expression alterations corresponding to certain pathological conditions. This integrated way is a useful tool to investigate molecular mechanism of diseases (7). Two dimensional polyacrylamide gel electrophoresis (2D-PAGE) enables separation of proteins with different molecular weight and isoelectric point (pI) (8). This method is widely used to perform functional proteomics (i.e., the large-scale analysis of alterations in protein expression levels) (9). Using 2D-PAGE to investigate various diseases has indicated that this method provides vital information regarding discovery of biomarkers and understanding the molecular mechanism of disease onset and development (10-12). Many studies have shown that 2D-PAGE can determine the differences between normal and ES proteomes of cells (13). Based on the above-mentioned points, this study aimed to evaluate the patterns of differentially expressed serum proteins in ES and PNES cases.

## Methods

***Study design and setting***

In this cross-sectional study, 4 patients with mesial temporal lobe epilepsy and 4 patients with PNES were selected from patients with history of recurrent seizures, who were admitted to epilepsy monitoring unit of Sina Hospital, Tehran, Iran, for proteomic analysis. The informed consent form was signed by all the patients before being recruited in the study. This study was approved by Ethics committee of Shahid Beheshti University of Medical Sciences (code: IR.SBMU.RETECH.REC.1397.289).

***Participants***

Patients with other medical, neurologic or psychiatric diseases (rather than seizure) or history of recent head trauma were excluded from the study. Moreover, using medications except anti-epileptic drugs (AEDs) or psychoactive drugs was considered an exclusion criterion. 

***Data gathering***

Age, gender, epilepsy duration and frequency, and the history of medications were recorded for all using a predesigned checklist. All the patients underwent V-EEG to capture enough habitual events. ES and PNES cases were differentiated based on V-EEG findings. The epilepsy type was determined by an expert neurologist (epileptologist) based on ictal and inter-ictal EEG findings and the seizures’ semiology. Venous blood samples were obtained from all the patients within 1 hour after habitual seizures. Serum was separated by centrifugation at 4000 rpm for 10 minutes and aliquots were stored at −80 °C. All the proteomics materials were obtained from GE Health Care Life Sciences and SERVA Company. We used 2-DE Clean-Up Kit (GE Healthcare) for proteome extraction from the two groups. Following the extraction, determination of protein concentration was done by 2-DE Quant Kit (GE Healthcare). The first dimension, isoelectric Focusing (14) separates proteins based on their pI. Before this step, immobilized pH gradient (IPG) strips were rehydrated for 8 hours. After that, IPG strips were equilibrated for 30 minutes at room temperature in equilibration solution (Serva Kit). In the next step, separation based on MW was performed by applying HPE Flattop Tower (horizontal electrophoresis) using 2D HPE™ Double-Gel 12.5 % Kit (Serva Company) for about 3.5 hours. After electrophoresis, the gels were stained using SERVA HPE™Coomassie® Staining Kit according to the protocol and then scanned using a calibrated GS-800 densitometer (Bio-Rad) scanner (20). Gel analysis was done using Progenesis Same Spots Software and 1.5-fold increase or decrease was used as the cut-off value. Analysis of image spots (proteins) that appeared on the gel was done based on the following steps: scanning the gel image, identifying protein spots and quantifying (evaluating the color intensity of spot), matching gels, data analysis, data interpretation and finally creation of two-dimensional electrophoresis databases (9). 

An epileptologist was responsible for data gathering.

***Statistical analysis***

We used χ^2^ test, analysis of variance (ANOVA), and least significant difference (LSD) Post Hoc test for statistical analysis. The data were analyzed by using SPSS-16 software. The findings were reported as mean ± standard deviation (SD), median (inter-quartile range; IQR); or frequency (%). The significance level was considered p< 0.05.

## Results

***Baseline characteristics of studied cases***

Based on V-EEG findings, 4 patients had mesial temporal lobe epilepsy (ES) and 4 patients had PNES. Table 1 shows the baseline characteristics of the studied cases. The mean duration of disease was 37.5 ± 7.76 years in ES group and 33.25 ± 18.7 years in PNES group. The mean frequency of seizure was in the range of 16.75 ± 15.30 attacks per month in the PNES group, and 3 ± 0.95 episodes per month in the ES group. MRI findings were normal in PNES group. All ES cases and 2 PNES cases were on poly-therapy. Only one patient in PNES group and none of those in ES group were on psychiatric medications (anti-depressants or neuroleptics).

***Proteomic analysis***

**Figure 1:**
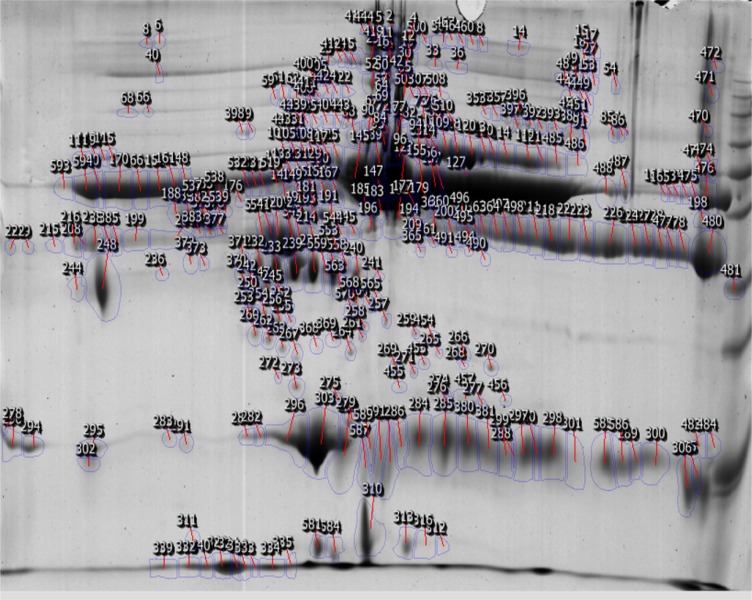
Position of matched spots on 2DE gel by comparing serum samples of epileptic seizure and psychogenic non-epileptic seizure (PNES) cases. The detected proteins were identified using SameSpots software

**Figure 2:**
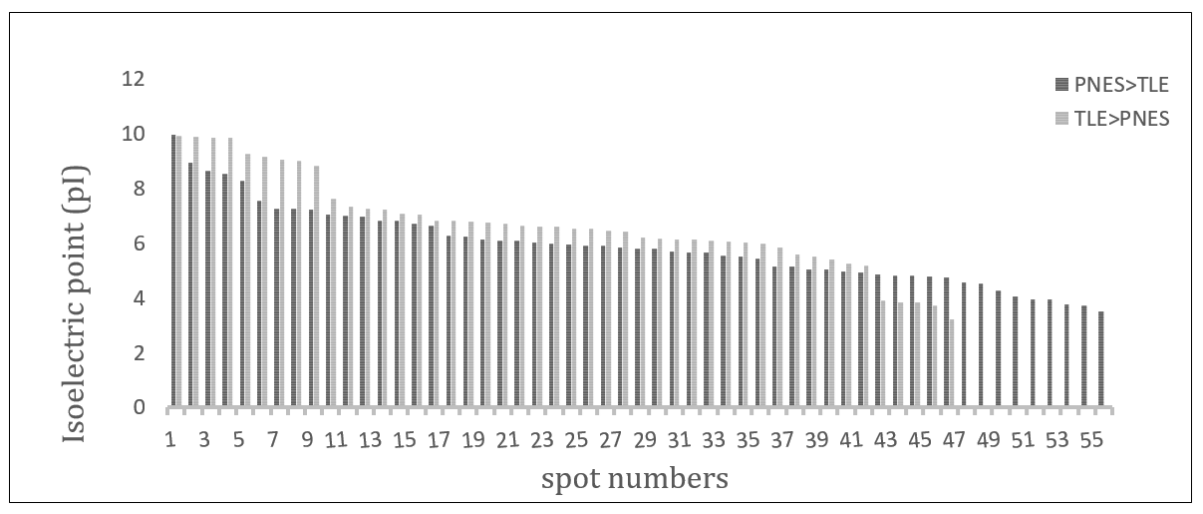
Isoelectric point (pI) distribution of proteins with 1.5-3.0 fold change in patients with temporal lobe epilepsy (TLE) and psychogenic non-epileptic seizure (PNES) (p = 0.008).

**Figure 3:**
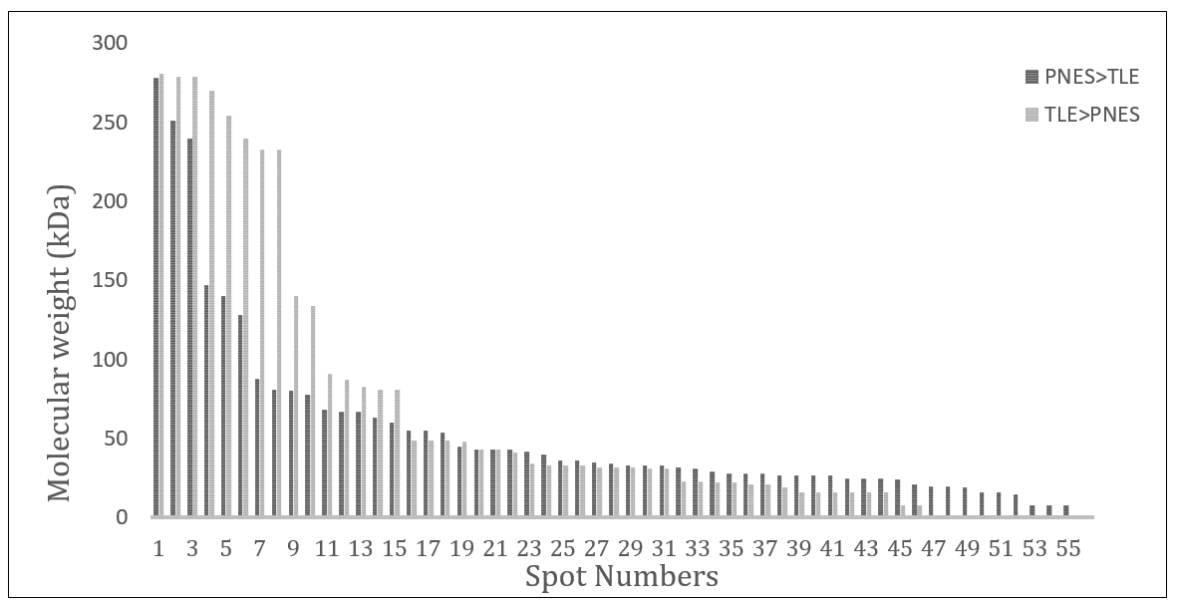
Molecular weight distribution of proteins with 1.5-3.0 fold change in patients with temporal lobe epilepsy (TLE) and psychogenic non-epileptic seizure (PNES) (p = 0.05).

As shown in the figure 1, 361 proteins were identified; of these, expression of 197 proteins had altered. 110 (55.9%) proteins were down-regulated and 87 (44.1%) were up-regulated in the PNES samples compared to ES samples. Distributions of isoelectric point (pI) and molecular weight fold changes for deregulated proteins with 1.5 to 3 fold changes in expression are presented in figures 2 and 3. The mean pI for deregulated proteins with 1.5 to 3 fold changes were 6.69 ± 1.68 in proteins with increasing expression in ES group and 5.88 ± 1.39 in proteins with increasing expression in PNES group (p = 0.008). The median and interquartile range (IQR) of molecular weight changes in proteins with 1.5 to 3 fold changes were 64 (IQR: 22.0-86.0) in proteins whose expression had increased in ES group and 39.5 (IQR: 26.0-61.5) in proteins whose expression had increased in PNES cases (p = 0.05). 

## Discussion

New diagnostic methods have been found over the past years to help diﬀerentiate ES from PNES. To date, many biomarkers have been assessed as potential candidates to diﬀerentiate ES from PNES, such as prolactin (PRL) (15, 16), cortisol (17, 18), neuron-speciﬁc enolase (NSE) (19), brain-derived neurotrophic factor (BDNF) (20), and Ghrelin and Nesfatin-1 (21) but until now no single biomarker has successfully differentiated PNES from ES; in fact, PNES is only diagnosed via the negation of ES (22) 

In this study, different expression of proteins between the two groups allows us to consider the idea of potential protein biomarkers for differential diagnosis of ES and PNES. Our findings, as depicted in figure 2, showed that up-regulated proteins with higher molecular weights were observed in temporal lobe epilepsy (TLE) group relative to the PNES samples. A similar pattern was obtained for pI values and up-regulated proteins in TLE had a higher pI value (figure 3).

In various neurological disorders, including epilepsy, the BBB (Blood Brain Barrier) is disrupted. Disruption of the blood brain barrier also contributes to epileptogenesis by facilitating the exposure of neurons to pro-inflammatory cytokines. It is also thought to play a role in drug resistance by becoming permeable to various transporters and enzymes (16). As a result of increased permeability in the BBB, the molecules that are normally expected to be found only in the central nervous system (CNS) may find a chance to diffuse into peripheral blood or on the contrary, serum proteins may reach the brain tissue. Some of these molecules are S100 calcium-binding protein β (S100β), neuron-specific enolase, glial fibrillary acidic protein (GFAP), and albumin (5). Other studies reported that nano bodies with a high pI 9/5 (23, 24) spontaneously cross the BBB. There are two main hypotheses for explaining this observation. First it may be a failure of the BBB. In this regard, small serum proteins with low pI value can penetrate the brain tissue, which leads to decrease in the serum level of these proteins. On the other hand, protein composition of serum is prominently made up of heavy proteins. The second hypothesis is that naturally, the heavy proteins with higher pI value are significantly deregulated in patients. The second hypothesis cannot be true because it is impossible for heavy proteins to be targeted naturally in the patients. As mentioned earlier, BBB damage is reported in ES patients. The gross alteration in permeability of BBB after ES promotion has been confirmed by several researches. Few proteomic studies have been done on epilepsy. Some authors have employed proteomic analysis to identify proteins that are differentially expressed in the hippocampus of patients with mesial temporal lobe epilepsy (MTLE) compared to control tissue obtained via autopsy. The researchers found that several proteins with different roles in the CNS were deregulated. The cytosolic enzyme acyl-CoA thioester hydrolase, known for its role in energy production via β-oxidation in mitochondria and peroxisomes, signal transduction, ion fluxes, and activation of protein kinase C, was down-regulated in hippocampus of patients with MTLE (25). In subsequent studies, these authors verified a decrease in expression of collapsing response-mediated protein- 2 (CMRP-2, 55 kDa protein), which is often involved in axonal outgrowth, path finding, and neuronal polarity processes (26). They also observed a decrease in expression of 18 proteins playing different roles in brain (25, 27). Danis et al. (28) performed a 2D-PAGE study, comparing GAERS (Genetic Absence Epilepsy Rat from Strasbourg) to non-epileptic control (NEC) rats. This study showed five differentially expressed proteins, two in the parietal cortex (ATP synthase sub- unit delta and the 14-3-3 zeta isoform), two in the thalamus (myelin basic protein and macrophage migration inhibitory factor (MIF)), and the other in the hippocampus (MIF and 0-beta 2 globulin). Almost all proteins were up-regulated in GAERS compared to NEC with the exception of 0-beta globulin. In line with this study, MIF was also found to be up-regulated in the frontal cortex and in the hippocampus of rats subjected to kainic acid-induced epilepsy (29). MIF is a pro-inflammatory cytokine released in response to inflammatory stimuli and is highly expressed in immune and non-immune cells, including those in the brain. A recent study by Conboy et al. (30) showed that MIF is important to the process of hippocampal neurogenesis, affecting cell proliferation in the dentate gyrus. In a recent study, by using proteomics (2D-PAGE), Persike et al. (13) showed that the total number of spots were noticeably smaller in the hippocampus of patients with pharmacoresistant TLE than in the control tissue. A total of 16 proteins were differentially expressed in the hippocampus of these patients compared to the control. However, only nine proteins were identified in this study. Among the nine deregulated proteins, six were up-regulated while one of them was down-regulated in the TLE group compared to controls. The other two proteins were only identified in the 2D-PAGE of epilepsy patients. In recent decades, new proteomics technologies have been developed to help us find appropriate protein biomarkers in serum. Simultaneous changes in many proteins in this disease can indicate biochemical mechanisms involved in its incidence, which can be effective for medicinal purposes in the treatment process. Few studies have measured and compared PI/MW of proteins whose expression significantly changed in temporal lobe epileptic patients and PI/MW in data base (31, 32). By measuring pI and molecular weight, Behboodi et al. reported that a more malignant cancer is associated with a more acidic pI and lower molecular weight for proteins detected in tissue (33). Further studies are needed to identify differentially expressed proteins between the two groups. 

**Table 1:** Baseline characteristics of patients with epileptic seizure (ES) and psychogenic non-epileptic seizure (PNES)

**Variables**	ES* Group (n = 4)	**PNES Group (n = 4)**
**Gender**		
Male	2 (50)	2 (50)
Female	2 (50)	2 (50)
**Age (years)**		
Mean ± SD	37.5 ± 7.76	33.25 ± 18.7
**Disease duration (years)**		
Mean ± SD	7.5 ± 4.20	17.2 ± 9.06
**Seizure frequency (per month)**		
Mean ± SD	3 ± 0.95	16.75 ± 15.30
**MRI finding**		
Left Hippocampus sclerosis	2 (50)	-
Right Hippocampus sclerosis	2 (50)	-
Normal	NA	4
**Medications**		
AED Mono-therapy	NA	1 (25 )
AED Poly-therapy	4 (100 )	2 (50 )
Psychoactive drugs	NA	1 (25 )

Data are presented as mean ± standard deviation (SD) or frequency (%). *: Patients with temporal lobe epilepsy. MRI: Magnetic resonance imaging; AED: Antiepileptic drug.

## Conclusion:

It can be concluded that blood brain barrier damage in epileptic seizures is the main event that can discriminate PNES patients from ES individuals. Therefore, identification of the deregulated proteins will provide a clear perspective of ES.
